# hERG S4-S5 linker acts as a voltage-dependent ligand that binds to the activation gate and locks it in a closed state

**DOI:** 10.1038/s41598-017-00155-2

**Published:** 2017-03-02

**Authors:** Olfat A. Malak, Zeineb Es-Salah-Lamoureux, Gildas Loussouarn

**Affiliations:** INSERM, CNRS, l’Institut du Thorax, Université de Nantes, 44007 Nantes, France

## Abstract

Delayed-rectifier potassium channels (hERG and KCNQ1) play a major role in cardiac repolarization. These channels are formed by a tetrameric pore (S5–S6) surrounded by four voltage sensor domains (S1-S4). Coupling between voltage sensor domains and the pore activation gate is critical for channel voltage-dependence. However, molecular mechanisms remain elusive. Herein, we demonstrate that covalently binding, through a disulfide bridge, a peptide mimicking the S4-S5 linker (S4-S5_L_) to the channel S6 C-terminus (S6_T_) completely inhibits hERG. This shows that channel S4-S5_L_ is sufficient to stabilize the pore activation gate in its closed state. Conversely, covalently binding a peptide mimicking S6_T_ to the channel S4-S5_L_ prevents its inhibiting effect and renders the channel almost completely voltage-independent. This shows that the channel S4-S5_L_ is necessary to stabilize the activation gate in its closed state. Altogether, our results provide chemical evidence that S4-S5_L_ acts as a voltage-controlled ligand that binds S6_T_ to lock the channel in a closed state, elucidating the coupling between voltage sensors and the gate in delayed rectifier potassium channels and potentially other voltage-gated channels.

## Introduction

Voltage-dependent ion channels are ubiquitously expressed in human tissues. They perform a plethora of physiological functions such as generation and modulation of the electrical activity in excitable tissues, modulation of neurotransmitter and hormone release, and electrolyte transport in epithelia. Canonical voltage-gated ion channels are tetramers of subunits containing six transmembrane segments (S1 to S6). Each of the four subunits is composed of one voltage sensor domain (S1 to S4) and a pore domain (S5-S6). The four pore domains tetramerize to generate a single pore module, which is regulated by the four voltage sensor domains^1, 2^. One key question remains: how do the voltage sensor domains regulate pore gating? Several independent approaches support the idea that interaction between the S4-S5 linker (S4-S5_L_) and the S6 C-terminal (S6_T_) part plays a critical role in that regulation^2–5^. First, Lu and collaborators pinpointed the sequence complementarity between S4-S5_L_ and S6_T_ in the Shaker channel^4, 5^. Second, structural data indicated the close proximity between S4-S5_L_ and S6_T_ and suggested a mechanical lever model to explain the coupling between the voltage sensor and the gate of the related K_v_1.2 channel^2^. However, the mechanism may prove to be more complex. Since 2006, several works on other eukaryotic K_v_ channels suggested state-dependent S4-S5_L_ and S6_T_ interactions^3, 6–10^, but the precise mechanism remains elusive and may vary from one channel to another.

**Figure 1:**
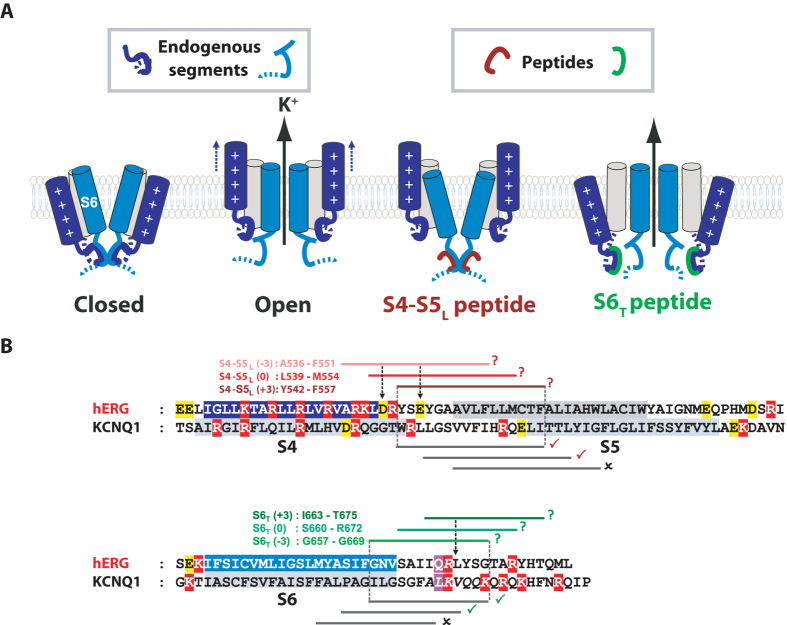
Hypothetical ligand/receptor model. Alignment used to design S4-S5_L_ and S6_T_ peptides. (**A**) Scheme of the hypothetical ligand/receptor model in which S4-S5_L_ (deep blue) binds to S6_T_ (light blue) to stabilize the channel in a closed state. Upon membrane depolarization, S4 pulls S4-S5_L_ out of the S6_T_ receptor, allowing channel opening. The S4-S5_L_ peptide (red) mimics endogenous S4-S5_L_, locking the channel in its closed conformation. Contrarily, S6_T_ peptide (green) binds to the endogenous S4-S5_L_ and limits its locking effect, leading to channel up-modulation. (**B**) Alignment used to design hERG peptides from previously identified KCNQ1 S4-S5_L_ and S6_T_ peptides (based on the multiple alignment obtained using Clustal Omega, presented in Supplemental Fig. 4). In red are represented the basic residues, in yellow acidic residues, and in purple the position of the narrowest part of the bundle crossing, also named the gating residue (see methods). The color boxes represent the transmembrane segments. Grey lines represent the peptides tested in KCNQ1 while red lines and green lines represent the designed hERG S4-S5_L_ and S6_T_ peptides, respectively. A check sign (✓) indicates that the KCNQ1 S4-S5_L_ peptide inhibits the channel (red) and that the KCNQ1 S6_T_ peptide activates the channel (green).

In a previous study, we suggested that the voltage dependence of the cardiac voltage-gated KCNQ1 channel (K_v_7.1) follows a ligand/receptor model. In this model, S4-S5_L_ is a ligand whose interaction with S6_T_ is only possible at resting potentials, and this interaction locks the channel in a closed state. Upon depolarization of the plasma membrane, S4 pulls the S4-S5_L_ out of its binding site and unlocks the channel (Fig. 1A). We validated this ligand/receptor model by using peptides mimicking S4-S5_L_ and S6_T_ and evaluating their effects on the channel activity. However the peptides effects were moderate, probably due to the low affinity between native S4-S5_L_ and S6_T_, which is necessary for S4-S5_L_ ligand unbinding and channel opening during membrane depolarization^11^. In the present study, we started with a similar strategy of peptide native-sequence mimicry to evaluate the ligand/receptor model in hERG (K_v_11.1), a key channel in cardiac and neuronal electrical activity. As in the KCNQ1 results, hERG-mimicking peptide effects were moderate. In a previous study on this channel, a disulfide bond was created between S4-S5_L_ and S6_T_, stabilizing it in a closed state^6^. Here, after the first step of peptide identification, we used the same cysteine approach to increase the peptide-channel interaction and to target the peptide to its hypothetical receptor (S6_T_ for S4-S5_L_ and *vice-versa*). We observed that covalently binding a peptide mimicking S4-S5_L_ to the endogenous S6_T_ could completely inhibit hERG. On the other hand, covalently binding a peptide mimicking S6_T_ to the endogenous S4-S5_L_ rendered the channel almost voltage-independent, most likely by preventing the inhibitory effect of S4-S5_L_ on S6_T_ in the channel.

Altogether, these results suggest that the voltage dependence of these two delayed rectifier channels, KCNQ1 and hERG, follows a ligand/receptor model in which S4-S5 linker acts as an inhibitor, locking the activation gate under the control of the voltage sensor S4.

## Results

### One S4-S5_L_ peptide inhibits hERG channels

To identify a potential inhibitory S4-S5_L_ peptide, three S4-S5_L_ encoding plasmids were designed based on sequence alignment with KCNQ1 (Fig. 1B), in which the ligand/receptor model was first suggested, the S4-S5 linker (S4-S5_L_) being the inhibiting ligand and the S6 C-terminus (S6_T_) acting as the receptor^11^. To study peptide effects on channel activity, each plasmid coding for one of the S4-S5_L_ peptides was co-transfected with a plasmid encoding the hERG channel.

**Figure 2:**
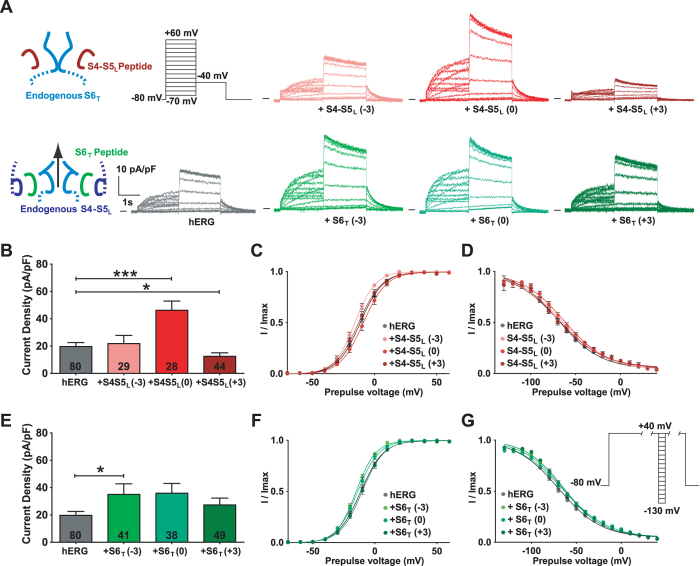
S4-S5_L_ (+3) peptide inhibits and S6_T_ (−3) peptide activates hERG channels. (**A**) Representative, superimposed recordings of the WT hERG current in the absence (0.6 µg hERG plus 1.4 µg GFP plasmids) and in the presence of S4-S5_L_ or S6_T_ peptides (0.6 µg hERG plus 1.4 µg peptide plasmids). Left: schemes of the hypothetical effects of S4-S5_L_ inhibiting or S6_T_ activating peptide; activation voltage protocol used (one sweep every 8 s) located above the WT hERG currents; right: data from experiments performed in the presence of various S4-S5_L_ or S6_T_ peptides. (**B**) Mean hERG tail-current density at −40 mV after a prepulse at +60 mV in the presence of S4-S5_L_ peptides. (**C)** Activation curve, obtained from tail currents using the protocol shown in A, in the presence of S4-S5_L_ peptides (n = 21–65). (**D**) Inactivation curve in the presence of S4-S5_L_ peptides (n = 6–24). Inset, Inactivation voltage protocol used (one sweep every 5 s, first pulse = 1 s, second pulse = 15 ms, third pulse = 0.5 s). (**E**) Through (**G**) same as B through D, in the presence of S6_T_ peptides (F, n = 28–65; G, n = 15–24). *p < 0.1, ***p < 0.01 *versus* control hERG, Mann-Whitney test.

**Figure 3:**
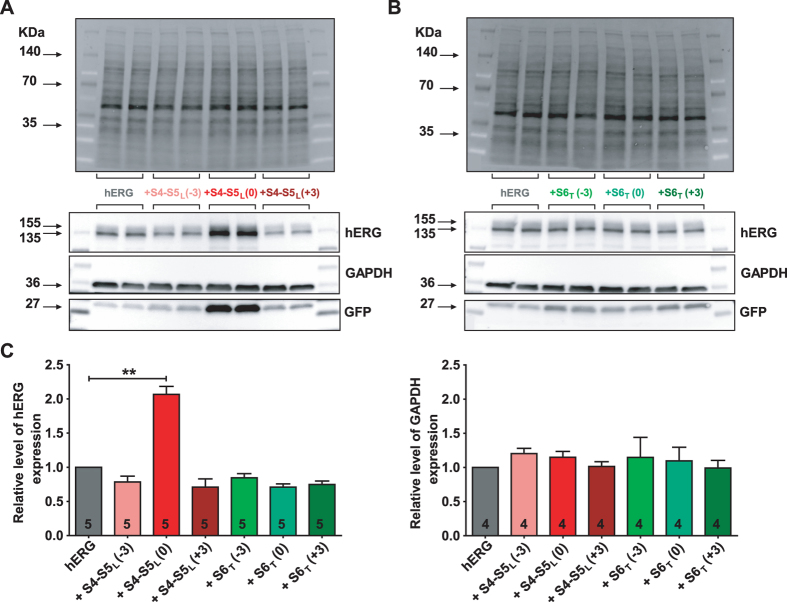
S4-S5_L_ (0) peptide increases hERG channel expression. (A,B) western blot analysis of hERG protein expression in COS-7 cell lysates in the absence or presence of S4-S5_L_ peptides (**A**) or S6_T_ peptides (**B**). Top: stain-free image of total proteins. Bottom: western blot images of hERG, GAPDH, and GFP proteins. The three blots, realized on the same membrane, are cropped. Full-length blots of each tested protein are reported in Supplemental Fig. 1. (**C**) Histogram of normalized mean intensity of hERG (left) and GAPDH (right) bands in the absence and in the presence of various S4-S5_L_ or S6_T_ peptides. Band intensities are first normalized to the intensity of the corresponding stain-free membrane lane, and ratios are then normalized to control hERG condition. **p < 0.01 *versus* control hERG, Mann-Whitney test realized on non-normalized ratios.

If the endogenous S4-S5_L_ acts like a ligand to inhibit the activation gate, then a peptide mimicking endogenous S4-S5_L_ should decrease hERG channel activity. As expected, the S4-S5_L_ (+3) peptide decreased the current density (Fig. 2A and B, Supplemental Table 1). Western blot experiments revealed that this decrease was not due to a lower expression of hERG protein in the presence of this peptide, as compared to control conditions (Fig. 3A and C). Thus, these results are consistent with the ligand/receptor model.

However, the S4-S5_L_ (−3) peptide did not show any effect on current density and led to a 5-mV negative shift in the activation curve (Fig. 2A–C, Supplemental Table 1). The S4-S5_L_ (−3) peptide presents 5 charged amino-acids, 3 positively charged on one face of the helix and 2 negatively charged on the other face. The absence of any effect on current density suggests a non-specific effect such as membrane charge screening, locally changing the potential detected by the voltage-sensor.

The S4-S5_L_ (0) peptide induced a paradoxical increase in hERG current density without any effect on the activation curve (Fig. 2A–C, Supplemental Table 1). Western blot experiments revealed that this peptide led to a doubling of hERG protein as compared to control conditions (Fig. 3A and C). Surprisingly, this peptide also induced an increase in GFP expression (by a factor of 7.1, p < 0.01), as shown in Fig. 3A. However expression of endogenous control GAPDH was not altered by S4-S5_L_ (0) (Fig. 3A and C).

This set of experiments shows specific effects of only one of the S4-S5_L_. The S4-S5_L_ (+3) peptide decreased the current density without modifying the activation curve, similar to the effects of the S4-S5_L_ inhibiting peptides on KCNQ1 channel^11^. This is consistent with the ligand/receptor model.

### One S6_T_ peptide activates hERG channels

If S6_T_ is the receptor for S4-S5_L_, then a peptide mimicking endogenous S6_T_ should act as a decoy to the endogenous S4-S5_L_, preventing its binding to the endogenous S6_T_ and increasing the channel activity. Indeed, the S6_T_ (−3) peptide up-regulated the WT channel and shifted the activation curve (and inactivation curve) by 5 mV toward negative potentials (Fig. 2A,E–G, Supplemental Table 1) without any change in the hERG expression level (Fig. 3B and C), which is similar to the effects of the S6_T_-activating peptides on KCNQ1 channel^11^ and therefore also consistent with the ligand/receptor model. The other peptides, namely S6_T_ (0) and S6_T_ (+3) did not produce any effect.

Altogether, the effects of one inhibiting and one activating peptide were similar for both hERG and KCNQ1 channels^11^. Moreover, both hERG inhibiting and activating peptides are aligned with those of the KCNQ1 channel (Fig. 1). In conclusion, these two similarities suggest that the ligand/receptor model applies to both channels.

### A cysteine disulfide bond reinforces the effect of the S4-S5_L_ (+3) peptide, leading to full inhibition of hERG channel

Although consistent with the ligand/receptor model, the S4-S5_L_ (+3) effect is moderate, as in KCNQ1, likely because the S4S-5_L_/S6_T_ interaction has to be loose, as explained in the introduction. In *Xenopus* oocytes, introduction of a cysteine pair in the hERG channel (D540C in S4-S5_L_ with L666C in S6_T_) has been previously shown to stabilize the channel in a closed conformation through the formation of a disulfide bond^6^. We reasoned that this disulfide bond could be exploited to covalently bind the mimicking S4-S5_L_ (+3) peptide to the S6_T_ channel, which should enhance its inhibitory effect.

**Figure 4:**
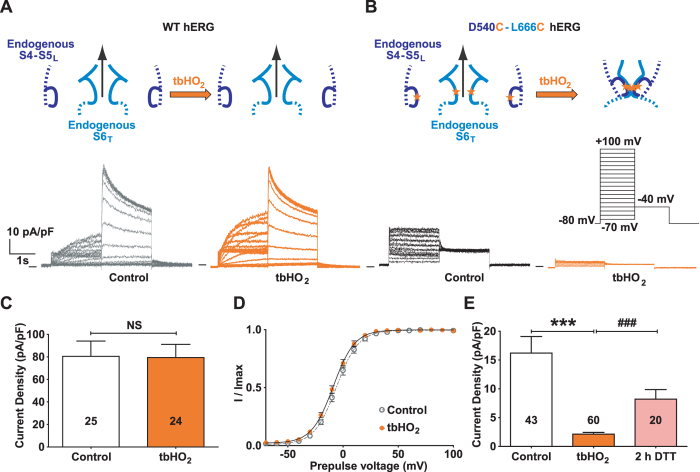
Introduction of 2 cysteines in the S4-S5_L_ and S6_T_ regions of hERG (D540C-L666C hERG) locks the channel closed in oxidative conditions. (**A**) Representative, superimposed recordings of the WT hERG current (3.6 µg plasmid plus 0.4 µg GFP plasmids) after 2 h incubation in Tyrode without (control) or with 0.2 mM tbHO_2_ (tbHO_2_). (**B**) D540C-L666C hERG channels, same as in A (3.6 µg plasmid plus 0.4 µg GFP plasmids). Cartoons: introduction of a cysteine is symbolized by a star. (**C**) Mean WT hERG tail-current density at −40 mV after a prepulse at +100 mV. (**D**) Activation curve obtained from the tail currents, using the protocol shown in (**B**). (**E**) Mean D540C-L666C hERG tail-current density at −40 mV after a prepulse at +100 mV, after 2 h incubation in Tyrode without (control) or with 0.2 mM tbHO_2_ (tbHO_2_), or after 2 h incubation in 10 mM DTT following tbHO_2_ incubation (2 h DTT). ***p < 0.001 *versus* control and ^###^p < 0.001 *versus* tbHO_2_, Mann-Whitney test.

First, we evaluated the effects of oxidizing the native cysteines on WT hERG activity. We observed that in COS-7 cells, as previously shown in *Xenopus* Oocytes, WT currents were unaffected after 2 hours of incubation with 0.2 mM tert-butylhydroperoxide (tbHO_2_; Fig. 4A,C and D, Supplemental Table 2), showing that oxidizing the native cysteines does not have any effect on channel gating and trafficking. Such a tbHO_2_ treatment led to a permanent closure of the D540C-L666C hERG mutant and this inhibition was reversed by subsequent incubation with 10 mM DTT (Fig. 4B and E, Supplemental Table 2). Finally, the current density of the single mutants D540C and L666C was not altered by incubation with tbHO_2_ (Supplemental Fig. 2), confirming that the D540C-L666C mutant inhibition by tbHO_2_ is specifically due to the disulfide bond formed between D540C and L666C.

**Figure 5:**
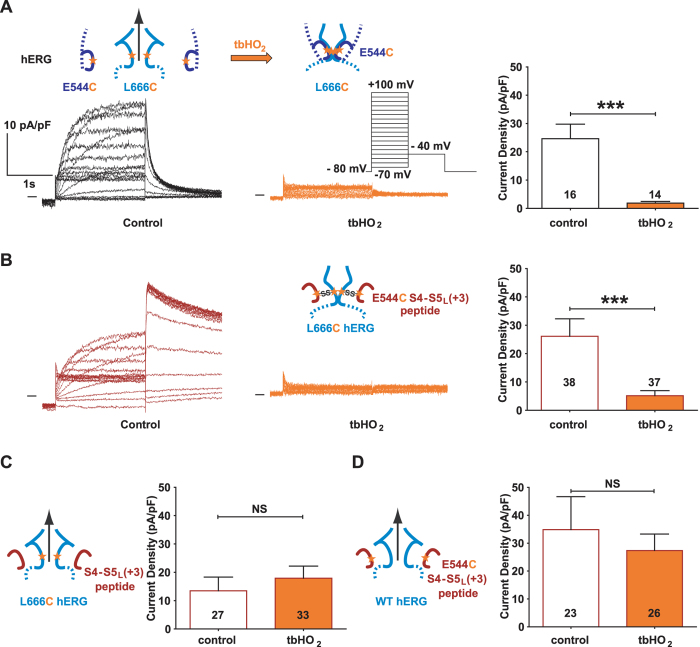
A cysteine disulfide bond reinforces the effect of the S4-S5_L_ (+3) peptide, leading to full inhibition of the channel. (**A**) left, representative, superimposed recordings of the E544C-L666C hERG current (3.6 µg E544C-L666C hERG plasmid plus 0.4 µg GFP plasmids) and right, mean tail-current density at −40 mV after a prepulse at +100 mV after 2 h incubation in Tyrode without (control) or with 0.2 mM tbHO_2_ (tbHO_2_), both using the voltage protocol shown in inset. (**B**) left, representative, superimposed recordings of the single mutant L666C hERG current in the presence of E544C S4-S5_L_ (+3) peptide (1 µg L666C hERG plus 3 µg peptide plasmids) and right, mean tail-current density  measured in the same conditions as A. (**C**,**D**) Mean tail-current density in the presence of only one cysteine, *i*.*e*. L666C hERG +S4-S5_L_ (+3) peptide (**C**) or WT hERG +E544C S4-S5_L_ (+3) peptide (**D**). ***p < 0.001 *versus* control, Mann-Whitney test.

The inhibiting S4-S5_L_ (+3) peptide does not contain the D540 residue (Fig. 1), but the E544 residue, located on the same side of the S4-S5_L_ helix as D540, is negatively charged as well. Both D540 and E544 may interact electrostatically with R665 (next to L666) in S6_T_ when the channel is in the closed state^12, 13^. Therefore, we tested if a cysteine introduced in E544 hERG instead of D540 and another cysteine introduced in L666 hERG could also form a disulfide bridge, which would lead to a permanent closure of the channel. COS-7 cells were transfected with the E544C-L666C double mutant hERG, and the generated current was indeed inhibited by incubation with 0.2 mM tbHO_2_, while the E544C and L666C single mutants were not (Fig. 5A, Supplemental Fig. 2). Since E544C/L666C can form a disulfide bond within the channel, we tested if this same cysteine pair could also covalently link the E544C mutated S4-S5_L_ (+3) peptide onto the S6_T_ of L666C single mutant hERG channel. Cells were co-transfected with the E544C S4-S5_L_ (+3) peptide and the L666C channel. Two hours of incubation with 0.2 mM tbHO_2_ almost completely inhibited the current (Fig. 5B, Supplemental Table 3), and this effect was specifically due to the presence of both cysteines (Fig. 5C and D, Supplemental Table 3). In conclusion, covalently binding S4-S5_L_ to S6_T_ inhibited the channel, strongly suggesting that S4-S5_L_ acts as a ligand binding to S6_T_ and locking the channel in a closed state.

### hERG inhibition provoked by E544C S4-S5_L_ (+3) peptide covalent binding to L666C mutant channel is reversible

**Figure 6:**
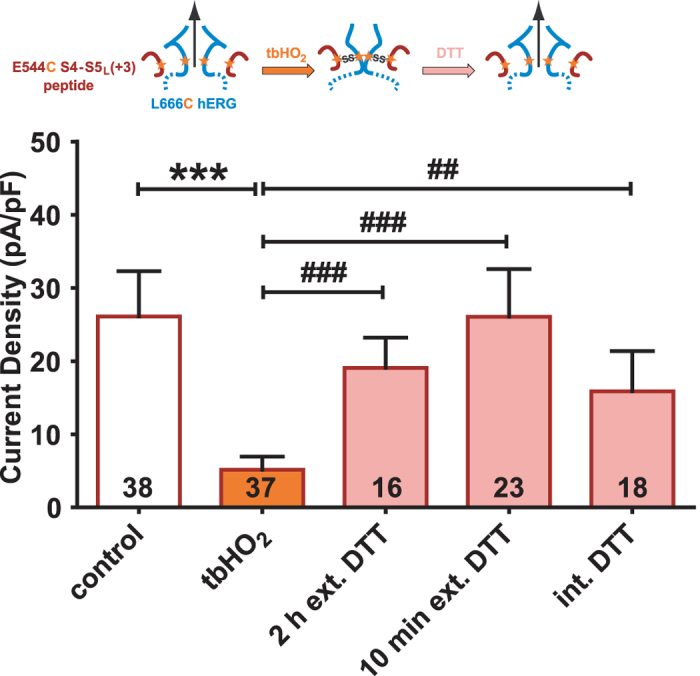
hERG inhibition provoked by E544C S4-S5_L_ (+3) peptide covalent binding to L666C mutant channel is reversible. Mean hERG tail-current density at −40 mV after a prepulse at +100 mV of L666C hERG channels in the presence of E544C S4-S5_L_ (+3) peptide (1 µg L666C hERG plus 3 µg peptide plasmids) after 2 h incubation in Tyrode without (control) or with 0.2 mM tbHO_2_ (control and tbHO_2_, respectively, same results as in Fig. 5B), and after 2 h or 10 min incubation in 10 mM DTT following tbHO_2_ incubation, or after direct application of 10 mM DTT in the pipette tip, following tbHO_2_ incubation. ***p < 0.001 *versus* control, ^##^p < 0.01 and ^###^p < 0.001 *versus* tbHO_2_, Mann-Whitney test.

Cells co-transfected with the E544C S4-S5_L_ (+3) peptide and the L666C channel were incubated for 2 hours with tbHO_2_, followed by 2 hours incubation with 10 mM DTT. This double treatment reversed the effect induced by tbHO_2_ incubation, as current densities were comparable to the control condition (Fig. 6). To exclude that the current recovery is due to *de novo* arrival of hERG at the plasma membrane during the 2 hours DTT incubation, we tested two additional conditions: 1) 10 mM DTT was applied for only 10 minutes or 2) we measured the current in the presence of 10 mM DTT in the pipette tip. In both conditions, current densities were similar to control, consistent with DTT application leading to peptide unbinding.

### hERG activation by S4-S5_L_ (0) peptide is not reinforced by its covalent binding through a disulfide bond

**Figure 7:**
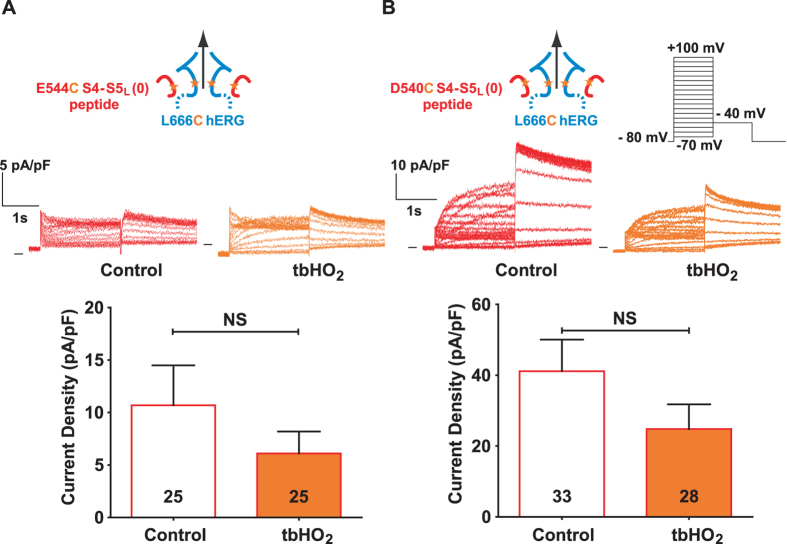
Unexpected hERG activation by S4-S5_L_ (0) peptide is not reinforced by its covalent binding though a disulfide bond. (**A**) Top, representative, superimposed recordings of the L666C hERG current in the presence of E544C S4-S5_L_ (0) peptide (1 µg L666C hERG plus 3 µg peptide plasmids) after 2 h incubation in Tyrode without (control) or with 0.2 mM tbHO_2_ (tbHO_2_), using the protocol shown in (**B**). Bottom, corresponding mean hERG tail-current density at −40 mV after a prepulse at +100 mV. (**B**) D540C S4-S5_L_ (0) peptide same as in A.

S4-S5_L_ (0) peptide increased WT hERG current density (Fig. 2A and B), but this effect is due to an increased expression of hERG protein, suggesting no effect of S4-S5_L_ (0) on channel gating. Consistent with this hypothesis, the current generated by COS-7 cells co-transfected with E544C S4-S5_L_ (0) peptide and L666C hERG channel was not increased upon incubation with tbHO_2_ (Fig. 7A, Supplemental Table 4). Since S4-S5_L_ (0) also includes the D540 residue (Fig. 1), we also tested the effect of D540C S4-S5_L_ (0) peptide on L666C single mutant hERG channel. Again, the current was not increased upon tbHO_2_ application (Fig. 7B, Supplemental Table 4). Altogether, these observations confirmed that the paradoxical activation effect initially observed when S4-S5_L_ (0) peptide was co-transfected with WT hERG channel was not due to an interaction of this peptide with the S6_T_ region of hERG.

### A cysteine disulfide bond reinforces the effect of the activating S6_T_ peptide, rendering the channel almost voltage independent

**Figure 8:**
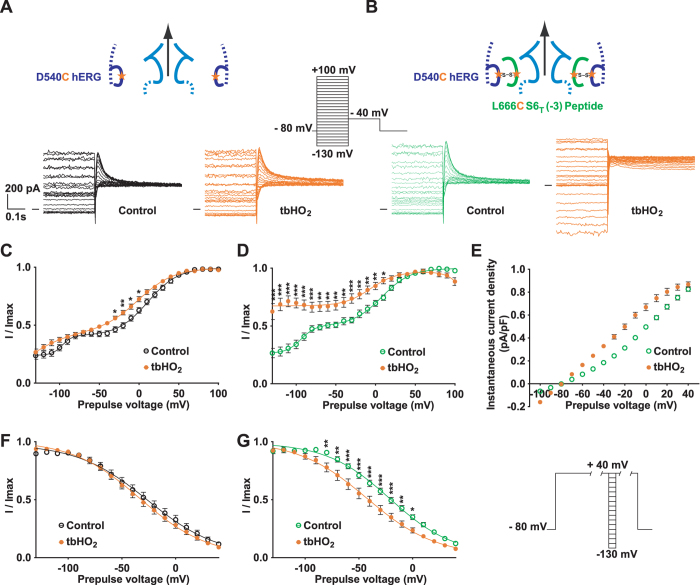
A cysteine disulfide bond reinforces the effect of the activating S6_T_ peptide, rendering the channel almost voltage independent. (**A**,**B**) Representative superimposed recordings of the single mutant D540C hERG current (**A**, 1 µg D540C hERG plus 3 µg GFP plasmids) and D540C hERG current in the presence of L666C S6_T_ (−3) peptide (**B**, 1 µg D540C hERG plus 3 µg peptide plasmids) after 2 h incubation in Tyrode without (control) and with 0.2 mM tbHO_2_ (tbHO_2_), using the voltage protocol shown in inset. (**C**,**D**) Activation curve, with a double Boltzmann, obtained from the tail currents in the same conditions as A, in the absence (**C**) or presence of the L666C S6_T_ (−3) peptide (**D**). (**E**) Maximum current density measured during the prepulse, using the protocol and conditions as in (**A**). (**F**,**G**) D540C hERG inactivation curve obtained using the protocol shown in right inset (same as in Fig. 2), in the absence (**F**) or presence of the L666C S6_T_ (−3) peptide (**G**). *p < 0.05, **p < 0.01, ***p < 0.001, two way ANOVA with Bonferroni test.

Since the S6_T_ peptide contains the L666 residue, we mutated this residue to a cysteine and tested the effect of this mutated peptide on the D540C single mutant hERG channel, both in the absence and in the presence of tbHO_2_. As shown in Fig. 8C, the D540C channel presents an atypical activation curve, with a double Boltzmann and a voltage-independent component, even at very negative potentials, unlike the WT channel. This suggests that the mutation D540C by itself alters the role of endogenous S4-S5_L_ as an inhibiting ligand. This voltage-independent component is similar to the one induced by some mutations in KCNQ1 that were interpreted as decreasing the interaction between S4-S5_L_ and S6_T_^11, 14^. Covalent binding of the L666C S6_T_ (−3) peptide to the D540C hERG channel dramatically increased the voltage-independent component (Fig. 8B and D). Such a tbHO_2_-dependent increase in the voltage-independent component was not observed in absence of the peptide (Fig. 8A and C). The voltage-independent component is potassium selective (Fig. 8E). These observations further confirm the ligand/receptor mechanism by demonstrating that S4-S5_L_ is necessary to lock the channel gate in a closed state.

Covalent binding of the L666C S6_T_ (−3) peptide to the channel D540C also leads to a 12-mV negative shift in the inactivation curve (Fig. 8G and Supplemental Table 5). In the hERG channel, inactivation is not directly coupled to activation^15, 16^, suggesting that the observed effect of the L666C S6_T_ (−3) peptide on the inactivation curve is not a consequence of a modification of activation. Of note, S6_T_ peptides bind to hERG S4-S5_L_ (Y542-F557) which is surrounded by regions that are involved in inactivation: double mutant cycle analysis suggests that interaction between L529, L532, V535 in S4 and I560, L564, I567 in S5 play a role in inactivation gating^17^. Thus, covalent binding of S6_T_ to S4-S5_L_ may alter this interaction leading to the observed effect on inactivation.

## Discussion

**Figure 9:**
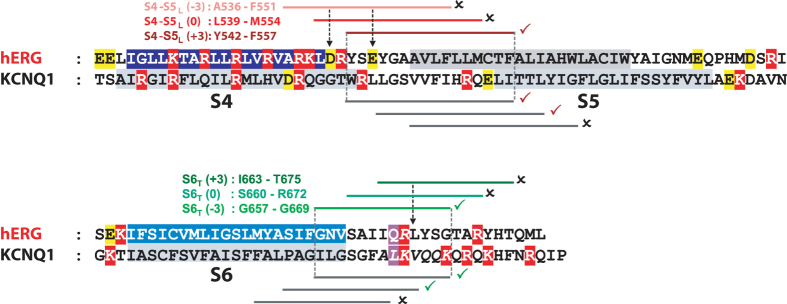
hERG inhibiting (S4-S5_L_ (+3)) and activating peptides (S6_T_ (−3)) are aligned with KCNQ1 inhibiting and activating peptides. Same as in Fig. 1B, onto which the results obtained with hERG have been added. A check sign (✓) indicates that the S4-S5_L_ peptide inhibits the channel and that the S6_T_ peptide activates the channel.

In a previous study on KCNQ1, and in the present study on hERG, we proposed a ligand/receptor model of voltage dependence. In this model, S4-S5_L_ would act as a ligand locking the S6_T_ gate in the closed state. To test this, we used S4-S5_L_ and S6_T_ mimicking peptides. Our results are in favor of this hypothesis. However, the effects were moderate. Here, we used another approach to reinforce the peptide effects on the hERG channel by introducing a cysteine pair covalently linking the mutated peptide to the single mutant channel. We observed that covalently linking the E544C S4-S5_L_ (+3) peptide to the L666C hERG almost completely inhibits the channel (Fig. 5B), by presumably preventing the outward splaying of the S6 helices. Covalently linking the L666C S6_T_ (−3) peptide to D540C hERG renders the channel almost completely voltage independent (Fig. 8D). We postulate that the covalently bound S6_T_ (−3) peptide blocks the normal interaction between endogenous S4-S5_L_ and S6_T_ which stabilizes the closed state, and thus the channel remains locked in the open state. For both peptides, either S4-S5_L_ or S6_T_, covalent link to the hERG channel clearly reinforces the respective effects of these two peptides when first tested on WT hERG channel without any introduced cysteine (as in Fig. 2). Altogether, these observations further reinforce the ligand/receptor model of voltage dependence. hERG S4-S5_L_ and S6_T_ peptides that produce the most potent effects are aligned with the most potent peptides of KCNQ1, strengthening the notion of a similar model for KCNQ1 and hERG activation gating (Fig. 9).

Cysteine introduction either in S4-S5_L_ or S6_T_ endogenous segments leads to alteration of the single mutant channel activity, as shown in Supplemental Fig. 3. This alteration is indicative of a change in the channel conformation. This observation may give rise to an alternative hypothesis: an artificial S4-S5_L_/S6_T_ interaction is only due to the cysteine introductions and does not exist in WT conditions. However, (i) S4-S5_L_ (+3) and S6_T_ (−3) peptides altered WT channel current density, suggesting that the ligand/receptor model is relevant for the WT situation even without the disulfide bond, (ii) despite the alteration of the channel voltage dependence due to the cysteines introduction, covalently linking the peptide to its target leads to effects which are consistent with the ligand/receptor model. Indeed, we observed, on one hand, a complete inhibition by E544C S4-S5_L_ (+3) on L666C hERG, and, on the other hand, a loss of voltage dependence by L666C S6_T_ (−3) on D540C hERG. In conclusion, two independent sets of experiments (with or without cysteines) are consistent with the ligand/receptor model. Interestingly, the same combination of cysteine introduction in S4-S5_L_ (0) peptide (E544C) and hERG (L666C), led to a complete loss of the current increase which was demonstrated to be nonspecific to the channel activity change but rather due to the channel expression increase when probed by western blot experiments. Altogether, our results suggest that cysteine introduction in the channel preserved the ligand/receptor mechanism that was originally observed in WT hERG (and also KCNQ1).

In the absence of structural data of K_v_ channels in the closed state, molecular mechanisms underlying the coupling between voltage-sensor movement and pore opening are still under debate. This coupling interaction has been suggested to be electromechanical and categorized as “attractive” or ”repulsive”, depending on the force exerted on the gate by the voltage sensors via the S4-S5_L_ and S6_T_ interaction^18^. In the “attractive” coupling, the S4 voltage sensors pull the gate to open it, requiring tight binding between S4-S_L_ and S6_T._ In the “repulsive” coupling, the voltage sensors push the gate to constrict it. In the present work on hERG channel, the ligand/receptor model corresponds to a third mechanism in which there is no need of a force directly linking the voltage-sensor and the gate, neither “attractive” nor ”repulsive”. Rather, S4-S5_L_ seems to act as a ligand that binds S6_T_ to lock the channel in a closed state. When S4 segments are in the “down” state at negative potential, S4-S5_L_ binds to S6_T_, locking the channel in the closed state. When S4 segments are in the “up” state, they pull S4-S5_L_ out of its binding pocket (S6_T_), unlocking the channel. This molecular mechanism is consistent with previous observations made on hERG channels and other K_v_ channels:

- Ferrer and collaborators showed that introduction of two cysteines, one in S4-S5_L_ and one in S6_T_, in the hERG channel, locks it in a closed state via a disulfide bond^6^. This is consistent with our hypothesis that S4-S5_L_ acts as an endogenous inhibitor, as confirmed by the exogenous peptide approach.
- Split hERG channels expressed as two distinct proteins, one containing the voltage sensor domain and one containing the pore, keep the same voltage dependence as the full-length channel^19^. In accordance with our model, where there is no mechanical force transduction from S4 movement to S6_T_ opening, there is no need for physical continuity between the two parts of the channel for activation transduction.
- The ligand/receptor model also helps in understanding why some hERG mutants, such as D540K, are open by both membrane depolarization and membrane hyperpolarization^20^. Since specific interactions between S4-S5_L_ and S6_T_ are needed to keep the channel closed, a charge reversal mutation on S4-S5_L_ may destabilize the interaction and lead to channel opening when S4-S5_L_ is pulled out of its S6_T_ binding pocket, either toward the cytosol (hyperpolarization) are toward the membrane (depolarization).
- Of note, it has been recently shown that the structure of the K_v_10.1 channel, which is 65% homologous to hERG, is incompatible with the mechanical lever mechanism^21^. In the open state structure of K_v_10.1, S4-S5_L_ is a short linker that is not domain swapped and, thus, is not in a position to function as a mechanical lever, leaving the possibility that the K_v_10.1 follows the same ligand/receptor model as hERG and KCNQ1.

This ligand/receptor model implies that when S4-S5_L_ is absent, the hERG activation gate should be in the open state, independent of the potential, as observed in one potassium^22^ and one sodium^23^ bacterial channels. In other words, this gate is intrinsically more stable in the open state. Surprisingly, hERG pore domain construct (S5-S6) alone, isolated from the voltage sensor domain, gives rise to a nonfunctional channel^19^. One particularity for hERG, compared to the aforementioned bacterial channels, is that the cytosolic N-terminus deletion greatly accelerates deactivation^24–26^. Thus, the N-terminus stabilizes the channel in the open state, and ablation of the hERG voltage sensor domain, which also deletes the N-terminus, may render hERG nonfunctional.

Of note, this ligand/receptor model is completely in frame with the allosteric model of voltage-dependence suggested for KCNQ1 and hERG, in which voltage-dependent movement of S4 is allosterically coupled to pore-opening transitions^27, 28^. In such a model, the gating of KCNQ1 can be well described by an allosteric model where the channel can open before all S4 voltage sensors have been activated^29, 30^. Our ligand/receptor model suggests that allosteric coupling is realized through the interaction between S4-S5_L_ and S6_T_ (leading to channel closure), which is favored when S4 segments are in the “down” state at resting potential and not favored when S4 segments are the in the “up” state.

In conclusion, the results that we obtained in the hERG channel with S4-S5_L_ and S6_T_ mimicking peptides, with or without cysteine introduction, reinforce the ligand/receptor model originally observed in KCNQ1 and suggest that this model applies to other voltage-gated channels. In Shaker channels, sequence complementarity between S4-S5_L_ and S6_T_ has also been shown to be critical to coupling voltage sensor movement and pore opening^4, 5^. Testing peptides mimicking S4-S5_L_ and S6_T_ in Shaker-like channels would confirm whether the ligand/receptor model applies to Shaker-like channels.

## Methods

### Plasmid constructs

Oligonucleotides encoding hERG peptides were synthesized by TOP Gene Technologies and contained a XhoI restriction enzyme, a methionine (ATG) for translation initiation, and a glycine (GGA) to protect the ribosome binding site during translation and the nascent peptide against proteolytic degradation^31^. A BamHI restriction enzyme site was synthesized at the 3′ end immediately following the translational stop codon (TGA). These oligonucleotides were then cloned into pIRES2-EGFP (Clontech) and sequenced.

Three different S4-S5_L_ and S6_T_ plasmids were designed with the same length (16 amino-acids for S4-S5_L_ and 13 for S6_T_) but different starting positions in order to compensate for the inaccuracy of the alignment due to the poor homology between hERG and KCNQ1 sequences (Fig. 1 and Supplemental Fig. 4). Multiple alignment was realized with Clustal Omega^32^. This program aligned the predicted transmembrane domains (Uniprot Q12809 for hERG, P51787 for KCNQ1) and the predicted position of the narrowest part of the bundle crossing, also named gating residue^33, 34^, of hERG and KCNQ1. We first designed a hERG S4-S5_L_ peptide based on the most potent KCNQ1 S4-S5_L_ (L251-L266^11^). Since mutagenesis studies suggested a close proximity of D540 in S4-S5_L_ and R665 in S6_T_^6, 13^, we chose two peptides shifted by 3 and 6 amino acids to the N-terminus to include the D540 residue. Names of the peptides were given according to their position along the sequence: S4-S5_L_ (−3) is A536-F551, S4-S5_L_ (0) is L539-M554, and S4-S5_L_ (+3) is Y542-F557. For S6_T_ peptides, we first selected the hERG sequence which aligned with the most potent peptide of KCNQ1 (I346-K358). Since in KCNQ1 the effects of the peptides were increased as the sequence was shifted to the C-terminus, we tried two other peptides position-shifted by 3 and 6 amino acids towards the C-terminus. S6_T_ (−3) is G657-G669, S6_T_ (0) is S660-R672, and S6﻿_T﻿_ (+3) is I663-T675.

### Cell culture and transfection

The African green monkey kidney-derived cell line COS-7 was obtained from the American Type Culture Collection (CRL-1651) and cultured in Dulbecco’s modified Eagle’s medium (GIBCO) supplemented with 10% fetal calf serum and antibiotics (100 IU/ml penicillin and 100 µg/ml streptomycin) at 5% CO_2_ and 95% air, maintained at 37 °C in a humidified incubator. Cells were transfected in 35-mm Petri dishes when the culture reached 50–60% confluence, with DNA (2 to 4 µg total DNA as described below) complexed with Fugene-6 (Roche Molecular Biochemical) according to the standard protocol recommended by the manufacturer. In the screening experiments used to identify the most potent peptides (Fig. 2), COS-7 cells were co-transfected with 0.6 µg pSI-hERG and 1.4 µg pIRES2-EGFP (Clontech) plasmids either encoding or not encoding a peptide. In the experiments with cysteine introduction, some mutations are associated with a decrease in current amplitude (D540C, E544C) or an increase in current amplitude (L666C). Plasmid quantities were thus optimized (i) to maximize the quantity of peptides, as assessed by the amount of florescence, and (ii) to keep the average current amplitude in the same range, in order to avoid undetectable currents or an incorrect voltage clamp. As a result, experiments on channels containing a D540C, E544C or L666C mutation were done with 1 µg mutated pSI-hERG and 3 µg pEGFP. Experiments on double mutant hERG channels (D540C/L666C or E540C/L666C) were performed with 3.6 µg pSI-hERG and 0.4 µg pEGFP. In pIRES2-EGFP plasmids, the second cassette (EGFP) is less expressed than the first cassette, guaranteeing high levels of peptides expression in fluorescent cells^11^. Cells were re-plated onto 35-mm Petri dishes the day after transfection for patch-clamp experiments.

### Western blot

20 µg of protein lysate from transfected COS-7 cells were fractionated on one-dimensional polyacrylamide gels and analyzed using western blotting with a goat polyclonal antibody against hERG (Santa Cruz Biotechnology, ref: SC-15968), a rabbit polyclonal antibody against GFP (Invitrogen Molecular Probes, ref: A11122), and a mouse monoclonal antibody against GAPDH (Santa Cruz Biotechnology, ref: SC-32233). Bound antibodies were detected using horseradish peroxidase-conjugated rabbit anti-goat (Santa Cruz Biotechnology, ref: SC-2922), goat anti-rabbit (Santa Cruz Biotechnology, ref: SC-2054), and goat anti-mouse (Santa Cruz Biotechnology, ref: SC-2055) secondary antibodies. Stain free gel technology (Bio-Rad) was used as loading control for protein normalization: band intensities were first normalized to the intensity of the corresponding stain free membrane lane, and ratios were then normalized to control hERG condition^35^.

### Electrophysiology

The day after splitting, COS-7 cells were mounted on the stage of an inverted microscope and constantly perfused by a Tyrode solution (cf. below) at a rate of 1–3 ml/min. The bath temperature was maintained at 22.0 ± 2.0 °C. Stimulation and data recording were performed with Axon pClamp 10 through an A/D converter (Digidata 1440A) using an Axopatch 200B (all Molecular Devices). Patch pipettes (tip resistance: 2–3 MOhms) were pulled from soda lime glass capillaries (Kimble-Chase). Currents were recorded in the whole-cell configuration. hERG activation and inactivation curves were obtained from the tail currents and fitted by Boltzmann equations.

### Solutions

The cells were continuously superfused with a HEPES-buffered Tyrode solution containing (in mmol/L): NaCl 145, KCl 4, MgCl_2_ 1, CaCl_2_ 1, HEPES 5, glucose 5, pH adjusted to 7.4 with NaOH. Patch pipettes were filled with the following solution (in mmol/L): KCl 100, Kgluconate 45, MgCl_2_ 1, EGTA 5, HEPES 10, pH adjusted to 7.2 with KOH. The membrane-permeable oxidizing agent tert-butylhydroperoxide (tbHO_2_) and dithiothreitol (DTT) were obtained from Sigma. Incubations  with tbHO_2_ and DTT were realized at room temperature.

### Statistics

All data are expressed as means ± S.E.M. Statistical differences between samples were determined using Student’s t-tests, rank-sum tests (when data were not normally distributed), and two-way analysis of variance associated with a Bonferroni post-hoc test when needed. In the screening experiment (Fig. 2 and Supplemental Table 1), a value of p < 0.10 was considered significant. In the rest of the study, a value of p < 0.05 was considered significant.

## Electronic supplementary material

Supplementary File
