# Supplementary material for: hERG S4-S5 linker acts as a voltage-dependent ligand that binds to the activation gate and locks it in a closed state
Source: Sci Rep. 2017 Mar 2;7:113. doi: 10.1038/s41598-017-00155-2 (PMC5427910; doi:10.1038/s41598-017-00155-2)
Supplement: Supplementary file 1 — Supplementary File [file 41598_2017_155_MOESM1_ESM.pdf]

hERG S4-S5 linker acts as a voltage-dependent ligand that binds to the activation gate and locks it in a closed state

Supplementary information

Olfat A. Malak[a], Zeineb Es-Salah-Lamoureux[a$], Gildas Loussouarn* [a$]

a. INSERM, CNRS, l'Institut du Thorax, Université de Nantes, 44007 Nantes, France

[$] ***These authors contributed equally to this work.***

Address correspondence to:
Dr. Gildas Loussouarn
L'institut du thorax
INSERM UMR 1087 / CNRS UMR 6291
IRS-UN, 8 Quai Moncousu BP 70721
44007 Nantes cedex 1, France
Tel: +33 (0)2 2808 0150
Fax: +33 (0)2 2808 0130
E-mail: gildas.loussouarn@inserm.fr

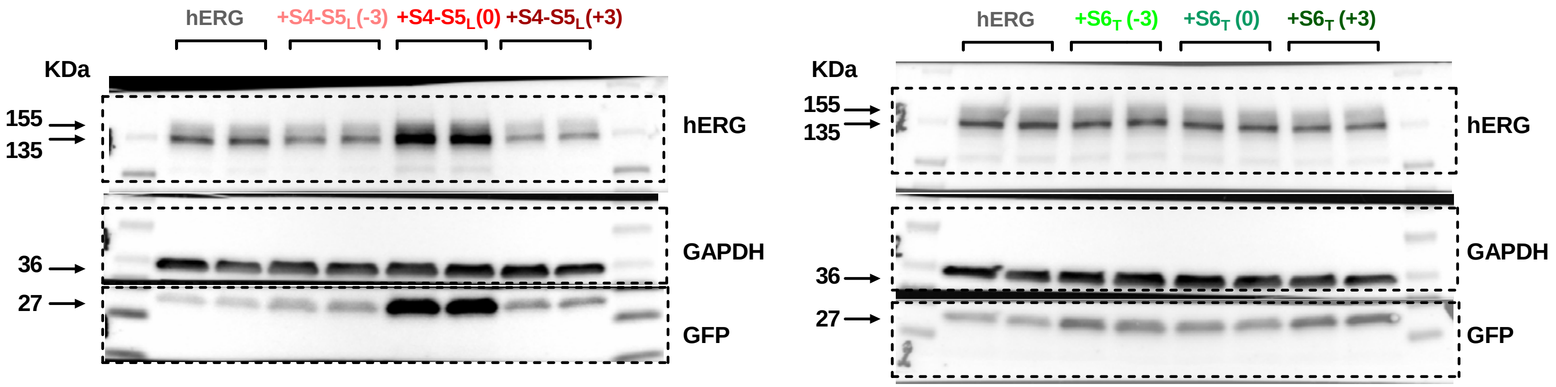

Supplemental Figure 1 : Full-length western blots corresponding to Figure 3. hERG, GAPDH, and GFP blots were realized on the same membrane. The membranes were cut in three pieces directly after the transfer. The dotted boxes represent the bands shown in the original figure.

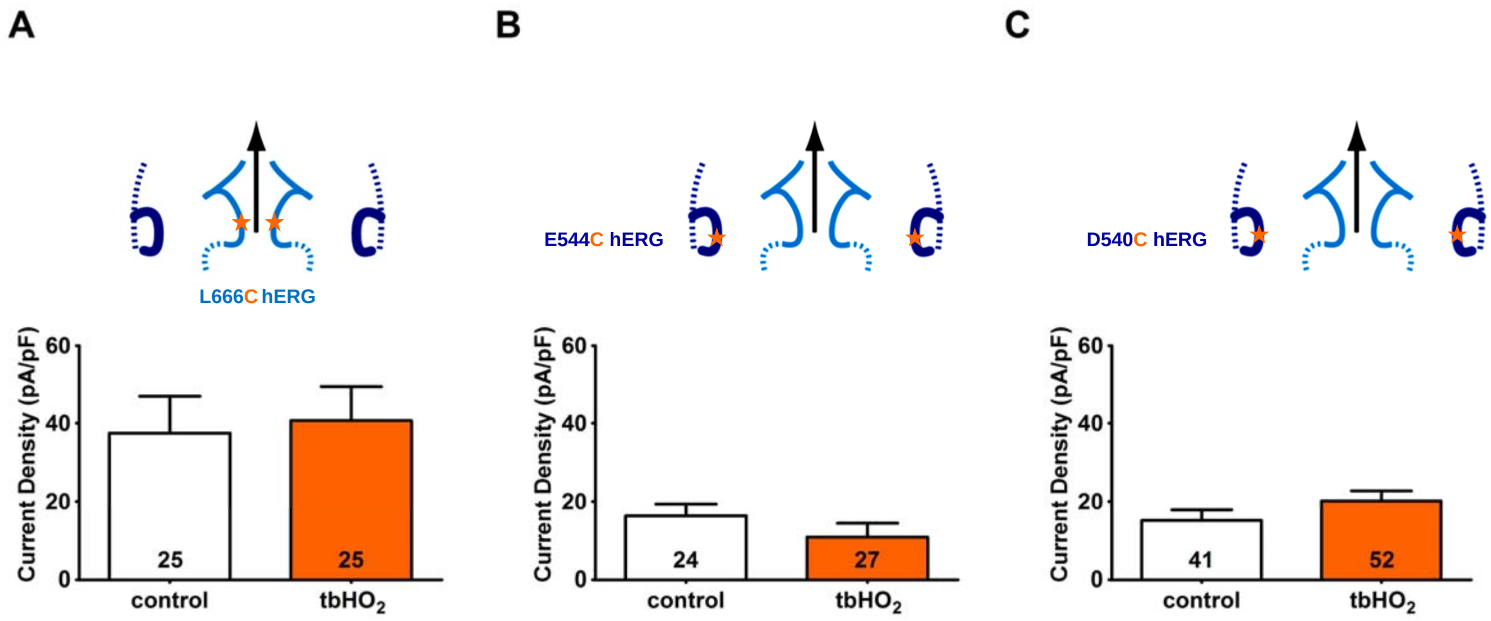

Supplemental Figure 2 : Single mutant controls. Mean hERG tail current densities at -40 mV after a prepulse at +100 mV of L666C (A), E544C (B) and D540C hERG channel (C), (1 µg of mutated channel plus 3µg of GFP plasmids) after 2h incubation in Tyrode without (control) or with 0.2 mM $tbHO_2$ ($tbHO_2$).

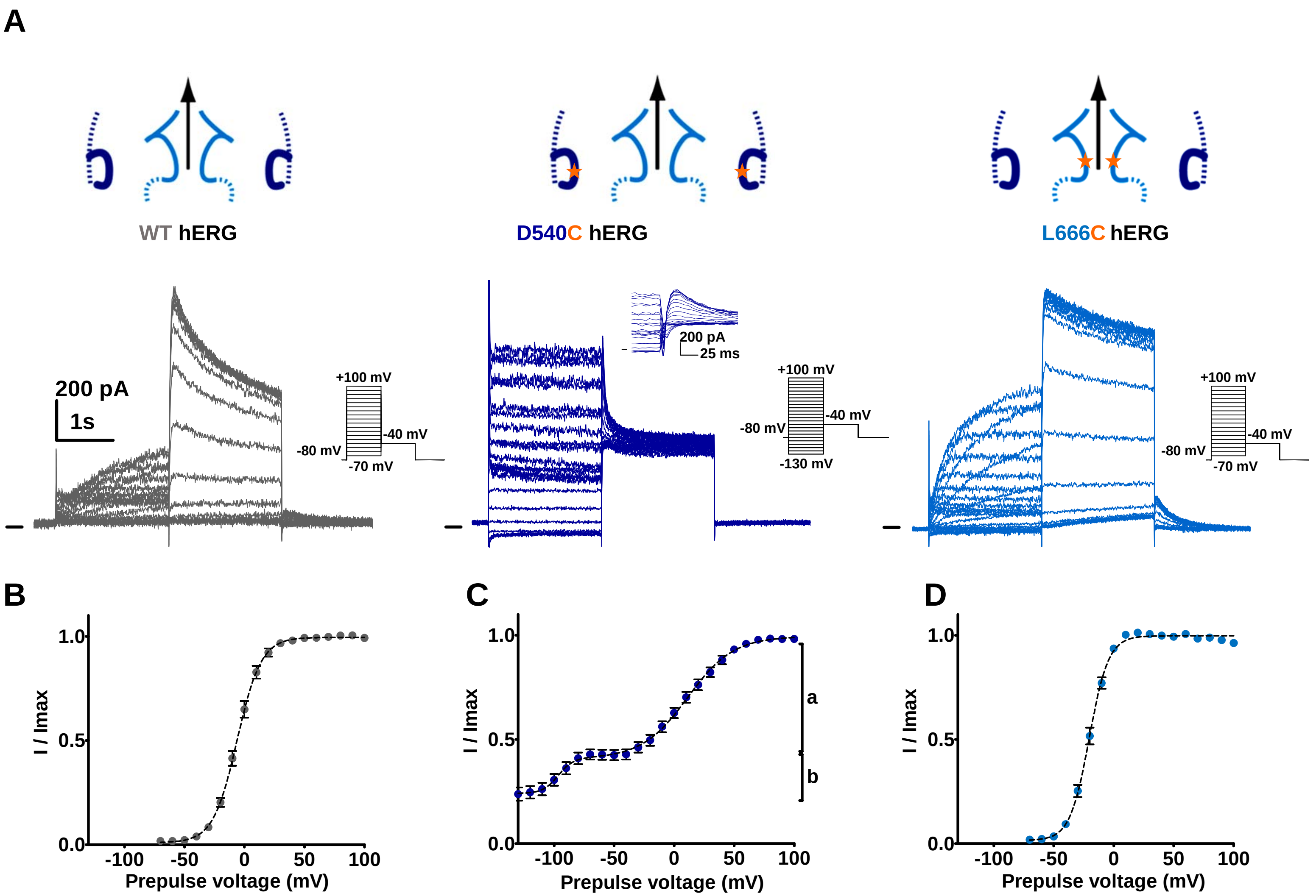

Supplemental Figure 3 : Activation curve of WT and single cysteine mutant hERG channels. **A:** representative, superimposed recordings of the WT (left), D540C (middle) and L666C (right) hERG current using the voltage protocols shown in inset. For D540C hERG, an inset shows a zoom-out of the tail current at -40mV. **B:** Activation curve of WT hERG obtained from the tail currents and fitted with a Boltzmann function ($V_{1/2}$= -5.6 ± 1.6 mV, K= 9.3 ± 0.6 mV, n= 20). **C:** Activation curve of D540C hERG obtained from the tail currents and fitted with a double Boltzmann function ($V_{1/2, a}$= 11.7 ± 2.3 mV, K_a= 18.1 ± 0.7 mV; $V_{1/2, b}$= -94.6 ± 1.5 mV, K_b= 7.7 ± 0.7 mV, n= 19). **D:** Activation curve of L666C hERG fitted with a Boltzmann function ($V_{1/2}$= -20.1 ± 1.2 mV, K= 7.5 ± 0.4 mV, n= 17).

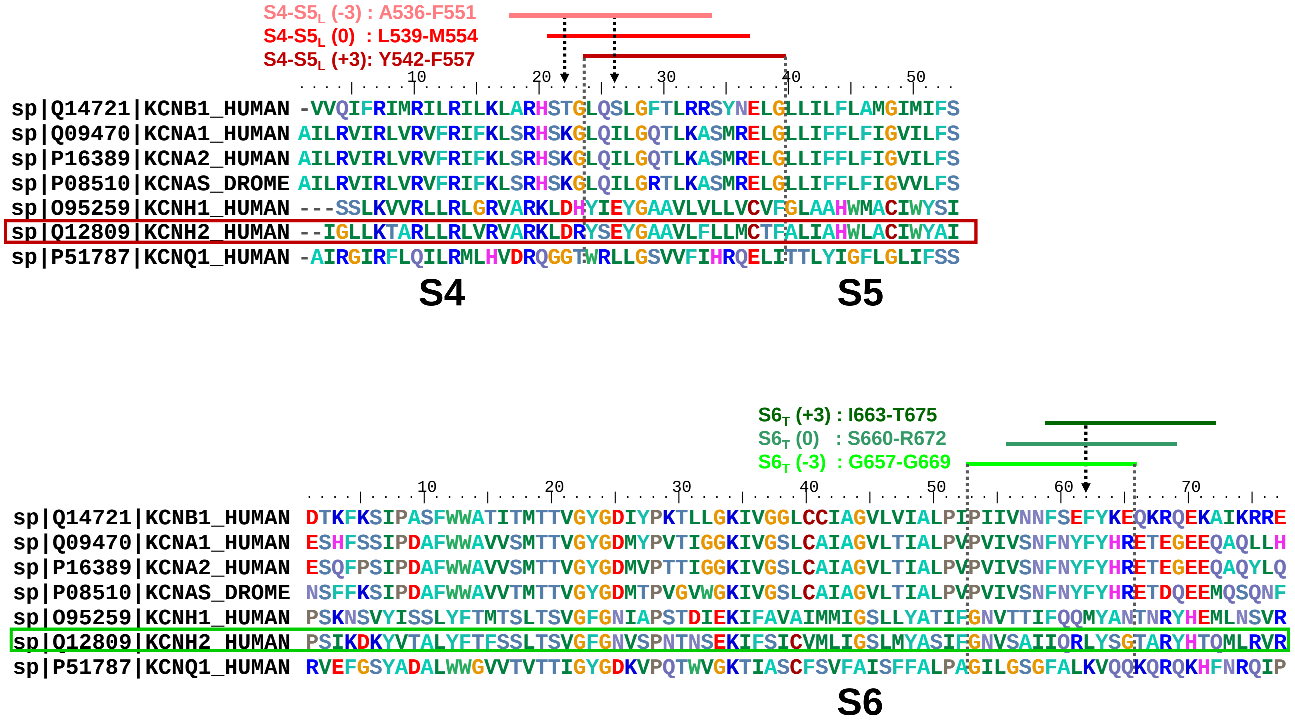

Supplemental Figure 4 : Multiple alignment of various human and D. melanogaster voltage-gated K+ channel sequences, showing poor homology between hERG (KCNH2) and KCNQ1 in the S4-S5$_L$ and S6$_T$ regions **(Clustal Omega)**.

	WT hERG	WT hERG + S4-S5$_L$ (-3)	WT hERG + S4-S5$_L$ (0)	WT hERG + S4-S5$_L$ (+3)	WT hERG + S6$_T$ (-3)	WT hERG + S6$_T$ (0)	WT hERG + S6$_T$ (+3)
**Current density (n)**	80	29	28	44	41	38	49
**at -40 mV (pA/pF)**	19.7 ± 2.9	21.8 ± 6	*** 46.3 ± 6.7	* 12.5 ± 2.5	* 35 ± 7.7	35.8 ± 7.1	27.3 ± 5
**Activation (n)**	65	21	21	33	34	28	38
**$V_{1/2}$ (mV)**	-10.9 ± 1.1	* -15.8 ± 1.1	-8.5 ± 1.7	-12.5 ± 1.5	* -15 ± 0.9	-13.7 ± 1.3	-10 ± 1.3
**K (mV)**	8 ± 0.2	7.9 ± 0.2	8.4 ± 0.3	8.2 ± 0.3	7.6 ± 0.3	7.8 ± 0.2	7.8 ± 0.2
**Inactivation (n)**	24	6	10	12	23	21	15
**$V_{1/2}$ (mV)**	-69.6 ± 1.9	-64.4 ± 3.2	-64.2 ± 2.8	-68.9 ± 2.6	* -65.2 ± 1.7	-65.6 ± 2.6	-65.2 ± 2.7
**K (mV)**	23 ± 0.6	25.1 ± 1.9	23.3 ± 0.8	25.5 ± 1.3	22 ± 0.4	24.3 ± 0.7	21.8 ± 0.6

Supplemental Table 1 : S4-S5$_L$ (+3) peptide inhibits and S6$_T$ (-3) peptide activates hERG channels. Mean hERG tail current densities at -40 mV after a prepulse at +60 mV, in the absence (WT hERG) or presence of S4-S5$_L$ inhibiting or S6$_T$ activating peptides (same transfection conditions as in Figure 2). Half activation/inactivation potential and slope of the activation/inactivation curves, obtained from tail currents, using the activation/inactivation protocol shown in Figure 2. *$p<0.1$, ***$p<0.01$ versus WT hERG in the absence of peptide, Mann-Whitney tests for current densities and Student's t-tests for activation and inactivation parameters ($V_{1/2}$ and K).

	WT hERG		D540C-L666C hERG		
$tbHO_2$	-	+	-	+	+
+ 2h DTT	-	-	-	-	+
Current density (n)	25	24	43	60	20
at -40 mV (pA/pF)	80.5 ± 13.6	79.5 ± 11.6	16.2 ± 2.9	*** 2.1 ± 0.3	### 8.2 ± 1.7
Activation (n)	20	18			
$V_{1/2}$ (mV)	-5.6 ± 1.6	-8.7 ± 1.4			
K (mV)	9.3 ± 0.6	9.3 ± 0.3			

Supplemental Table 2 : Introduction of 2 cysteines in S4-S5$_L$ and S6$_T$ of hERG (D540C-L666C hERG), locks this channel closed through a disulfide bridge in oxidative condition. Mean WT and D540C-L666C hERG tail current densities and WT hERG activation biophysical parameters in the same conditions as in figure 4. Same statistics as in figure 4E.

	E544C-L666C hERG		L666C hERG + E544C S4-S5$_L$ (+3)		L666C hERG + S4-S5$_L$ (+3)		WT hERG + E544C S4-S5$_L$ (+3)	
**$tbHO_2$**	**-**	**+**	**-**	**+**	**-**	**+**	**-**	**+**
**Current density (n)**	16	14	38	37	27	33	23	26
**at -40 mV (pA/pF)**	24.6 ± 5.2	*** 1.9 ± 0.6	26.1 ± 6.2	*** 5.6 ± 1.8	13.4 ± 4.9	17.9 ± 4.3	34.9 ± 11.8	27.3 ± 5.9

Supplemental Table 3 : A cysteine disulfide bond reinforces the effect of the S4-S5$_L$(+3) peptide, leading to full inhibition of the channel. Mean hERG tail current densities in the same conditions as in figure 5. Same statistics as in figure 5.

	L666C hERG		L666C hERG + E544C S4-S5$_L$ (0)		L666C hERG + D540C S4-S5$_L$ (0)	
**tbHO$_2$**	**-**	**+**	**-**	**+**	**-**	**+**
**Current density (n)**	25	25	25	25	33	28
**at -40 mV (pA/pF)**	37.5 ± 9.5	40.7 ± 8.7	10.7 ± 3.8	6.1 ± 2.1	41.1 ± 8.9	24.8 ± 7

Supplemental Table 4 : Activation by S4-S5$_L$(0) peptide does not occur through a S4-S5$_L$/S6$_T$ interaction. Mean hERG tail current densities at -40 mV after a prepulse at +100 mV of the single mutant L666C hERG channel alone in the same conditions as in supplemental figure 2A or in the presence of E544C or D540C S4-S5$_L$(0) peptide in the same conditions as in figure 7. *p<0.05 *versus* L666C hERG alone, Mann-Whitney tests.

	D540C hERG		D540C hERG + L666C $S6_T$ (-3)	
**$tbHO_2$**	**-**	**+**	**-**	**+**
**Inactivation (n)**	11	15	14	9
**$V_{1/2}$ (mV)**	-28.9 ± 6.1	-32.8 ± 5	-22 ± 3.5	** -43.8 ± 6.8
**K (mV)**	30.7 ± 0.6	* 28.7 ± 0.5	28.7 ± 0.6	28.7 ± 0.9

Supplemental Table 5 : Covalent binding of L666C $S6_T$(-3) onto the D540C hERG shifts the inactivation curve. Inactivation biophysical parameters in the same conditions as in figure 8G. *p<0.05, **p<0.01 *versus* without $tbHO_2$, Student's t-tests.
